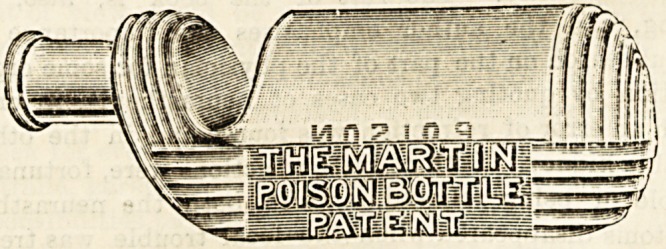# New Appliances and Things Medical

**Published:** 1902-09-20

**Authors:** 


### NEW APPLIANCES AND THINGS MEDICAL.

### PREPARATIONS OF THE PEPTENZYME CO.

(Peptenzyme Co., 80 Gloucester Road, South Kensington, London.)

The preparations known as peptenzyme probably require little introduction to the medical profession; their efficient action as artificial digestants are already known to the scientific world. It may, however, not be out of place to remind the readers of The Hospital that peptenzyme contains the ferments necessary for complete digestion in what we may call a nascent form. Peptenzyme is in fact composed of the enzyme, or mother-cells, of the ferment as they exist in the living glands, and their digestive properties are called into being the moment the conditions are suitable for ferment activity. In most cases of dyspepsia and impaired digestion Peptenzyme will be found more efficacious and reliable in its action than the ordinary ferments, pepsin and pancreatine. Lymocide, a new antiseptic and detergent liquid of non-poisonous properties, is also manufactured by the same firm, and deserves attention for all purposes in which a general antiseptic is required, alike for interal and external use. In some respects this antiseptic resembles listerine, containing a number of antiseptic oils, thymolate of soda, menthol, sulphocarbolate of zinc, and other powerful germicides and oxidising agents. It possesses an agreeable odour, and is particularly suitable as a mouth-wash, or for cleansing the nasal or aural cavities. Trophonine, a new liquid food of palpable and nutritious character, is also manufactured by the Peptenzyme Co. It is composed of vegetable and animal proteids combined with the enzymes of the digestive glands and the nucleo-albumins of the lymphoid tissues, thus constituting a liquid which is at once a stimulant a food and an adjuvant to digestion. Its use is indicated in all cases of nervous prostration, and especially in those in which the powers of digestion are impaired.

### MARTIN POISON BOTTLE,

(Surgical Supply Association, Ltd., 35 Market Street, Manchester.)

This new bottle, which is intended to be used for the dispensing of poisonous liniments, embrocations, lotions, and liquid disinfectants, fulfils the requirements of the Poison Act—that is to say, it can at once be recognised by sight and distinguished by touch alone from ordinary medicine bottles. The accompanying illustration represents the shape of the bottle, and in addition to the obvious advantages of its design, it is further in its favour that it cannot stand upright and that its contents do not escape if the cork is accidentally left out. It is practically sold at the same price as ordinary dispensing bottles. We commend this bottle to the notice of hospital, asylum, and dispensary authorities.

## Figures and Tables

**Figure f1:**